# Detection of Enterotoxigenic Psychrotrophic Presumptive *Bacillus cereus* and Cereulide Producers in Food Products and Ingredients

**DOI:** 10.3390/toxins14040289

**Published:** 2022-04-16

**Authors:** Jelena Jovanovic, Svitlana Tretiak, Katrien Begyn, Andreja Rajkovic

**Affiliations:** 1 Department of Food Technology, Safety, and Health, Ghent University, Coupure Links 653, 9000 Ghent, Belgium; jelena.jovanovic@ugent.be (J.J.); katrien.begyn@ugent.be (K.B.); 2 Department of Pathobiology, Pharmacology and Zoological Medicine, Ghent University, Salisburylaan 133, D5 Ingang 78, 9820 Merelbeke, Belgium; svitlana.tretiak@ugent.be; 3 Impextraco nv, Wiekevorstsesteenweg 38, 2220 Heist-op-den-Berg, Belgium

**Keywords:** *Bacillus cereus*, enterotoxins, cereulide, psychrotrophic growth, refrigeration temperature

## Abstract

In the last decade, foodborne outbreaks and individual cases caused by bacterial toxins showed an increasing trend. The major contributors are enterotoxins and cereulide produced by *Bacillus cereus*, which can cause a diarrheal and emetic form of the disease, respectively. These diseases usually induce relatively mild symptoms; however, fatal cases have been reported. With the aim to detected potential toxin producers that are able to grow at refrigerator temperatures and subsequently produce cereulide, we screened the prevalence of enterotoxin and cereulide toxin gene carriers and the psychrotrophic capacity of presumptive *B. cereus* obtained from 250 food products (cereal products, including rice and seeds/pulses, dairy-based products, dried vegetables, mixed food, herbs, and spices). Of tested food products, 226/250 (90.4%) contained presumptive *B. cereus*, which communities were further tested for the presence of *nheA*, *hblA*, *cytK-1*, and *ces* genes. Food products were mainly contaminated with the *nheA B. cereus* carriers (77.9%), followed by *hblA* (64.8%), *ces* (23.2%), and *cytK-1* (4.4%). Toxigenic *B. cereus* communities were further subjected to refrigerated (4 and 7 °C) and mild abuse temperatures (10 °C). Overall, 77% (94/121), 86% (104/121), and 100% (121/121) were able to grow at 4, 7, and 10 °C, respectively. Enterotoxin and cereulide potential producers were detected in 81% of psychrotrophic presumptive *B. cereus*. Toxin encoding genes *nheA*, *hblA*, and *ces* gene were found in 77.2, 55, and 11.7% of tested samples, respectively. None of the psychrotrophic presumptive *B. cereus* were carriers of the cytotoxin K-1 encoding gene (*cytK-1*). Nearly half of emetic psychrotrophic *B. cereus* were able to produce cereulide in optimal conditions. At 4 °C none of the examined psychrotrophs produced cereulide. The results of this research highlight the high prevalence of *B. cereus* and the omnipresence of toxin gene harboring presumptive *B. cereus* that can grow at refrigerator temperatures, with a focus on cereulide producers.

## 1. Introduction

*Bacillus cereus* sensu lato (s.l) is a group of Gram-positive, rod-shaped, motile, facultative anaerobes, mostly beta-hemolytic, catalase-positive, spore formers composed of a growing list of novel species which are widely distributed in the environment [1,2,3,4,5,6,7]. Officially, *Bacillus anthracis*, *Bacillus cereus* sensu stricto (s.s.), *Bacillus thuringiensis*, *Bacillus cytotoxicus*, *Bacillus weihenstephanensis*, *Bacillus mycoides*, *Bacillus pseudomycoides*, and *Bacillus toyonensis* are validated, and 12 more species have been described [1,2,3,4,5,6,7]. Genetically, these species are highly related [8]; however, *B. cereus* group members show diverse phenotypical characteristics. *B. anthracis* is a well-known zoonotic agent due to extreme pathogenicity, *B. toyonensis* is used as a probiotic, *B. thuringiensis* is an entomopathogen characterized by the insecticidal crystals and used as a bio-pesticide. In contrast, human foodborne pathogens are not strictly related to one species. Toxigenic *B. cereus* strains are widespread among different species and are identified as the causal agents of gastrointestinal diseases [9].

Two types of gastrointestinal diseases are caused by *B. cereus* toxins: diarrheal and emetic disease. The former is associated with enterotoxin(s) production in the gastrointestinal tract by ingested enterotoxigenic *B. cereus* strains. The latter is caused by emetic *B. cereus* strains, which produce cereulide in the food and ingredients during production or storage [10]. Although enterotoxins can be produced in food before ingestion, they typically do not reach the small intestine in sufficient amounts due to sensitivity toward low pH and digestive enzymes (e.g., pepsin) present in the upper parts of the gastrointestinal tract [11]. To induce food poisoning, enterotoxigenic *B. cereus* vegetative cells or spores have to be ingested in high counts, presumably corresponding to levels of 10^5^ CFU/g. However, concentrations as low as 10^3^ CFU/g have been implicated in cases of both diarrheal and emetic diseases [1]. *B. cereus* enterotoxins of the highest relevance to food safety are non-hemolytic enterotoxin (Nhe), hemolysin BL (Hbl), and cytotoxin K (CytK). Nhe and Hbl are three-component proteinaceous toxins encoded on the *nheABC* and *hblACD* operons. Both Nhe and Hbl are cytotoxic only in the presence of all three components (NheA, NheB, NheC and Hbl-B, Hbl-L1, and Hbl-L, respectively). CytK is composed of a single protein that exists in two forms, encoded by *cytK-1* and *cytK-2* genes, showing 89% of sequence similarity [12,13]. CytK-1 has been associated with several reported death cases, while CytK-2 is considered less cytotoxic. The second type of *B. cereus* foodborne disease, known as the emetic form, is caused by the thermo- and acid-stable cyclic peptide cereulide, encoded by the *ces* gene locus [14]. Usually, after consumption of food contaminated with cereulide, the disease manifests with emesis for up to five hours [15]. In high doses, cereulide can enter systemic circulation, cross the blood-brain barrier, cause fulminant liver failure, and damage other organs [16,17]. The effects are dose-dependent, but sensitive individuals, such as children, can suffer fatal consequences [10,18,19]. 

Up to date, several fatal cases and many outbreaks have been attributed to *B. cereus* foodborne poisoning [1,18,20,21,22,23]. Fatal cases and family outbreaks were usually caused by the intake of cereulide via mixed food products that contained farinaceous ingredients [16,18,20,23]. However, mild outbreaks and individual cases were related to many food types, including cereals/cereal products, herbs, spices, dairy products, confectionery products, canned food, seafood, vegetables, egg products, drinks, and dried food products [24,25,26,27,28]. The reason for such broad prevalence is that *B. cereus* is widespread in the environment (in dust, ground, plant surfaces, or in the rhizosphere, enteric tract insects, and mammals) and concomitantly in ingredients and final food products. The ability to form spores under unfavorable conditions enables *B. cereus* to survive, spread, and resist mild food processing and preservation treatments [29]. Moreover, mixing food ingredients of different microbiological quality in which intrinsic conditions are favorable for spore germination and vegetative outgrowth can change the overall microbiological picture of the newly formed product. Therefore, it is not surprising that mixed food was the most common cause of *B. cereus* food poisoning in the past [24,30,31]. Contaminated ingredients were the most important contributory factor reported for the strong-evidence outbreaks, together with the storage time/temperature abuse along the food production chain in the Europe Union (EU) in 2014 [25]. 

In food safety, a particular concern is given to *B. cereus* members that do not show uniform temperature growth affinities. Most strains are characterized by typical mesophilic properties, whose growth can be controlled by refrigeration. However, a subset of these mesophiles display as cold-tolerant (psychrotrophic) bacteria that can multiply at a temperature lower than 10 °C (at 7 and 4 °C). In this regard, both enterotoxin and cereulide producing psychrotrophic strains represent a microbial hazard in refrigerated food. Although significant amounts of cereulide cannot be produced at temperatures lower than 8 °C, temperature abuse is observed in each part of the cold chain, particularly in households of consumers, where the average temperature often surpass 8 °C [18,20,21,32,33]. 

In the last decade, *B. cereus* food poisonings outbreaks and individual cases showed an increasing trend, and in 2020, as the most frequently reported cause of food poisoning outbreaks in the EU [24,25,26,27,28]. Considering that *B. cereus* might induce diseases with fatal consequences, understanding *B. cereus* prevalence in food products and its ability to grow at refrigerator temperatures is an essential prerequisite to assure food safety. Therefore, the aim of this study was to estimate the prevalence of toxigenic *B. cereus* organisms among selected food products and ingredients, as well as the growth properties of *B. cereus* grown at refrigerator (4 and 7 °C) and mild abuse temperatures (10 °C). Additionally, the ability of psychrotrophic *ces*-positive presumptive *B. cereus* to produce cereulide at 37 °C and 4 °C was analyzed.

## 2. Results and Discussion

### 2.1. Distribution of Toxin Genes among Presumptive B. cereus Organisms Obtained from Food Products

*B. cereus* is one of the most common causal agents of foodborne diseases among bacterial toxin producers. Reports published by the European Food Safety Agency (EFSA) and European Center for Disease Control (ECDC) demonstrated that many food types were vehicles in strong-evidenced foodborne outbreaks [1,26,27,34,35]. In this study, we collected 250 food products and ingredients from the Belgian, Dutch and Serbian retail markets with the aim to estimate the prevalence of contaminated food products by the toxigenic *B. cereus*, toxigenic profiles of psychrotrophically grown presumptive *B. cereus* communities, and their ability to produce cereulide. In total, 226/250 (90.4%) food products contained presumptive *B. cereus*, which we further tested for the presence of *nheA*, *hblA*, *cytK-1,* and *ces* genes. Overall, selected food products were mainly contaminated with the *nheA* positive *B. cereus* carriers, found in 77.9% of the samples (176/250). Typically, *nhe* positive strains are the most prevalent among *B. cereus* food isolates [36,37,38,39,40]. However, the prevalence of the *nhe* gene among *B. cereus* food isolates reported in literature depends on the food type, as some studies indicated that the *hbl* gene was the most prevalent [41,42]. In our study, data showed that *hblA* positive presumptive *B. cereus* were found in 64.8% (162/250) of collected food samples. Also, Wijnands et al. [37] reported that *hbl* was detected in approximately 66% of the food isolates. Something higher proportion of Hbl-producing strains (766/1022), was reported by Berthold-Pluta et al. [38] in products collected from the Polish market (74.9%). Biesta-Peters et al. [39] found that nearly half of the food isolates contained the *hbl* genes. 

Further, in our study, 23.2% (58/250) of examined food products were contaminated with emetic *B. cereus* (Table 1). Similarly, around 17% of food isolates obtained from the Dutch market were classified as emetic strains [39]. However, not all emetic strains were able to produce cereulide, indicating that toxin encoding genes do not always express their pathogenic potential. In our study, we observed that among psychrotrophic *ces*-positive presumptive *B. cereus* producers 5 out of 11 were able to produce cereulide (Section 2.4). Further, 10 out of 250 products were contaminated with *cytK-1* positive presumptive *B. cereus* (4.4%). Generally, *cytK-1* positive isolates belong to *B. cytotoxicus* species, which is not highly prevalent. In France, 5% of isolates among presumptive *B. cereus* were identified as the carriers of the *cytK-1* gene, which is in line with our results [43]. Initially, *cytK-1* was detected in the strain that induced several death cases [12] and for a long time it was considered that all carriers of this gene are highly cytotoxic. However, discoveries of the novel strains indicate that cytotoxicity among *B. cytotoxicity* is very diverse, and therefore should be further examined [44,45]. 

In the category of cereal products, including rice and seeds/pulses (*n* = 162), *nheA* carriers were found in 73.4%, *hblA* in 68.5%, while *cytK-1* was found in 0.6% of examined products (129, 108, and 1/162, respectively). Potential emetic producers were detected in 20.37% (33/162) of tested products in this group. Specifically, in the subcategory of cereal grains, *nheA* was detected in almost 80% of presumptive *B. cereus* isolated from food (39/49). Buckwheat, millet, bulgur, quinoa, and rice were evidenced as a source of potentially toxin-producing *B. cereus*. Also, an experiment conducted on the flax grain produced in Canada showed that a large proportion of the *B. cereus* isolates carried the *nheA* (96.33%), *hbID* (94.5%), *cytK-1* (12.8%), while only 2.8% strains carried *ces* gene [46], indicating that cereal grains are an important source of potentially toxin-producing *B. cereus*. Further, raw rice is usually highly contaminated with *B. cereus*. Samapundo et al. [47] reported that 100% of tested rice marketed in Belgium was contaminated by presumptive *B. cereus*. We noted that the majority of tested raw rice samples harbored at least one toxigenic *B. cereus* (27/29). *nheA* carriers were the most prevalent (26/29), followed by *hblA* (21/29) and emetic *B. cereus* (5/29). Berthod-Pluta et al. found that 81.4% of isolates obtained from rice were able to produce Nhe, 57.6% Hbl, and 6.8% were *ces*-positive [47]. In rice obtained from the U.S. market, 89.1% of *B. cereus* isolates possessed the *nheAB* genes [48], and almost 53.0% of tested isolates were able to produce both Nhe and Hbl enterotoxins. Overall, 93.9% of those isolates were determined as pathogenic [48], but emetic strains were not found. 

Subcategory of cereal-based products and derivatives (*n* = 94) harbored *nheA* positive *B. cereus* in 70% (66/94) and *hblA* in 67% (63/94), respectively, while *ces* carriers were detected in 21.3% (20/94) of tested products. One sample in this group, namely couscous (*n* = 5), was contaminated with the all tested toxigenic *B. cereus*. Kone et al. [49] reported that only one cereal-based product (improved millet flour) out of 210 was contaminated with *B. cytotoxicus*, indicating that cereals do not represent the main source of *B. cytotoxicus*.

The high prevalence of toxigenic *B. cereus* among cereals is not surprising. Cereals and cereal-based products (including seeds and rice) were determined as common sources of *B. cereus* food poisonings [1]. Nevertheless, cereals and cereal-based products are usually used as ingredients in mixed food or ready-to-eat products [24,25,26,31], indicating that they might be a significant contributory source of *B. cereus*. Several death cases were linked with cereulide production in ready-to-eat pasta and rice-based products [16,18,20,21,27]. Therefore, in this study, we also examined ready-to-eat products that contain pasta and rice. Based on the results obtained in our study, neither the ready-to-eat pasta nor rice-based products (*n* = 12) were contaminated with emetic strains. *hblA* and *nheA* positive *B. cereus* were found in two and 4/12 tested products, respectively, from which 2/12 we concomitantly contaminated with both toxin gene carriers. Yu et al. [50] reported that 35% of ready-to-eat products were positive for *B. cereus*, from which 39 and 83% of the isolated strains contained enterotoxin-encoding *hblACD* and *nheABC* genes, respectively, while 7% harbored the *cesB* gene. Similarly, in India, ready-to-eat products were mainly contaminated with *nheABC* genes (57.6%), followed by 36.8% of *hblC* positive *B. cereus* [51]. Ready-to-eat products are usually mild heat-treated, which enables bacterial spores to survive. These mild treatments inactivate the vegetative flora; however, they may trigger the germination of present spores. Depending on temperature growth affinities, intrinsic and extrinsic environmental factors, a growing population can produce cereulide or enterotoxins during refrigeration [15,52]. Furthermore, we tested instant soups (*n* = 13) constituted from starch and spices. Results showed that these products are largely contaminated with presumptive *B. cereus* (11/13). *nheA*, *hblA*, were identified in samples obtained from 11 soups, and *ces* was also found in six products. Similar observations were reported by Messelhäusseret et al. [53]; however, instant soups were not extensively examined in the past.

Milk powder has been a known source of *B. cereus* for many years [54,55], as the processing of milk into a powder does not eliminate *B. cereus* spores [56]. In our study, the toxin gene harboring presumptive *B. cereus* were highly prevalent in dairy-based powders. In total, 22/26 products were contaminated by the *nheA*, followed by *hblA* positive *B. cereus* (22/26). Although *ces* positive *B. cereus* s.l. are mainly related to starch-rich products, in 10 products belonging to dairy-based powders, potential cereulide producers were detected. Ten products from this group (10/26) were positive on *nheA*, *hblA*, and *ces B. cereus* carriers together. Liu et al. [57] reported that 75% of isolates were *nha* positive, 21% *hbl* positive, while *ces* encoding genes were not detected. Also, our study showed that other milk powder analogs, such as coffee creamers and chantilly cream represent the source of potentially toxin-producing *B. cereus*. One out of seven coffee creamers analyzed in this study were contaminated with *nheA*, *hblA,* and *ces* positive *B. cereus* together. In five products, *nheA* and *hblA* positive *B. cereus* were found combined, and one product was contaminated with the *nheA* gene carrier. Also, three products of chantilly cream obtained from the Belgian market were tested. Two products were contaminated in the combination of *nheA*, *hblA*, and *ces* positive *B. cereus*. Generally, the microbiological safety of dried milk powders depends on the initial level of contamination of raw milk, processing parameters, and hygiene in the production area. The main concern is related to reconstructed milk powder-based infant formulas, which in case of temperature abuse might cause infection in infants and babies due to the lack of competitive intestinal flora.

Dehydrated vegetable products such as mashed potato flakes are often contaminated with *B. cereus* s.l. In our study, *nheA* positive *B. cereus* were the most dominant contaminants of mashed potato flakes (*n* = 17). *hblA* positive *B. cereus* was found in nine products, *ces* carriers in seven products, and *cytK-1* carriers were found in six products. Mashed potatoes flakes represent the most significant reported source of *cytK-1* positive strains, considering that it has been rarely found in other products. Kone et al. [49] reported that 20/20 potato flakes were contaminated with *B. cytotoxicus*. Heini et al. [58] also found that mashed potato powder is the most significant source of the *cytK-1* positive isolates. More considerably, our study showed that this product could be concomitantly contaminated with *nhe*, *hbl*, *ces*, and *cytK-1* positive *B. cereus* together (2/17). 

Spices (*n* = 12) and herbal teas (*n* = 8) also harbored potentially toxin-producing *B. cereus*. Among spices, 6/12 were contaminated with *hblA* positive *B. cereus*, while *nheA* positive *B. cereus* was found in 2/12 products. Six, four, and one tea product were contaminated with *nheA*, *hblA,* and *ces* carriers, respectively. Dry herbs and spices are well known to be contaminated with *B. cereus*. Indirectly, as ingredients in mixed food products, they can contribute to *B. cereus* food poisoning outbreaks and cases.

### 2.2. Growth of Toxigenic Presumptive B. cereus at 4, 7, and 10 °C

Psychrotrophic capacity varies within and among *B. cereus* species, as numerous lineages have been shown to grow at low temperatures. The principal food safety issue related to spore formers, such as *B. cereus* is the ability to survive the processing and preservation treatments and multiply during refrigerator storage to a concentration that might be infectious. Therefore in this study, we exposed toxigenic presumptive *B. cereus* communities obtained from different food products and ingredients (*n* = 121) to refrigerated (4 and 7 °C) and mild abuse temperatures (10 °C) for one month. In total, 94, 104, and 121/121 of presumptive *B. cereus* communities isolated from food products were able to grow at 4, 7, and 10 °C, respectively (Table 2). As expected, at 4 °C growth detection time (GDT) was the longest. The GDT at 4 °C ranged from 1–23 days, with an average of 11 days and a median of 15 days. In the first five days, 16/94 of presumptive *B. cereus* were detected to grow. The GDT for 7/94 and 11/94 ranged between 6–10 days and 11–15 days, respectively. The majority of tested *B. cereus* (32/94) grew in the range of 16–20 days, while for 18 tested *B. cereus*, the GDT was between 20–25 days. Day 23 was the last day when *B. cereus* was detected at 4 °C. 

It was not possible to determine a correlation between specific food groups and the presence of psychrotrophic presumptive *B. cereus* as psychrotrophs were highly prevalent in all food categories. In the category of cereal products, including rice and seeds/pulses, *B. cereus* was detected in a total of 48/70 of tested samples. Food products typically linked with *B. cereus* severe food poisonings, such as pasta and rice were also a source of psychrotrophic *B. cereus* (6/11 and 6/9, respectively). *B. cereus* obtained from powdered dairy-based products could also grow at 4 °C (7/7). Psychrotrophic presumptive *B. cereus* communities were found in coffee creamer (6/8) and chantilly cream (3/3), indicating that milk powder analogs are also a great source of psychrotrophic *B. cereus* able to grow at refrigerator temperature. Also, other products showed as a potential source of toxigenic presumptive *B. cereus* that can grow at refrigerator temperatures, such as mashed potato flakes (9/10), instant soups (12/12), and spices (6/7). 

Although our study demonstrated that psychrotrophic *B. cereus* are highly prevalent among different food products, data from the literature is diverse. For example, Samapundo et al. [47] showed that among 380 isolates, none were able to grow at 5 °C in 45 days. Similar results were reported by Park et al. who isolated *B. cereus* from the green lettuce [59]. Isolates obtained from cooked chilled products did not grow at 4 °C; however, 10% of these strains were able to grow at 5 °C [60]. Carlin et al. reported that 9/83 of isolates obtained from food and environment were able to grow at 4 °C [61]. *B. cereus* obtained from condiments were all psychrotrophic [62]. Currently, there is no standardized assay for defining *B. cereus* temperature growth affinities. Factors such as selection of media, growth conditions (i.e., temperature, agar plate vs. broth, shaking vs. non-shaking) represent potential sources of variation among data. While some researchers have suggested usage of the “psychrotolerant signatures” obtained via cold shock protein (encoded by *cspA*) or *panC*, this approach is still questioned as some non-psychrotolerant strains also encode these “psychrotolerant signatures” or are able to grow at low temperatures but do not possess any of the genes that would indicate there temperature growth affinities [63,64]. Based on the differences in 16 s rRNA, Wijnands et al. [30] reported that 4.4% of food isolates were psychrotrophic. Furthermore, researchers estimated psychrotrophic profiles by exposure of isolates at 6 °C. Isolates obtained from food products in Morocco had ability to grow at 6 °C (37/52) [65]. Also, Stenfors and Granum found that 17/26 were able to grow at 6 °C among dairy, food, and clinical isolates [66]. Jan et al. [64] reported that all isolates from egg products multiplied at 6 °C. Similarly, Torkar and Seme [67] detected 56.7% of *B. cereus* food and clinical isolates at 6.5 °C.

At 7 °C, 104/121 of presumptive *B. cereus* were detected. The GDT range and average were similar to results obtained at 4 °C (1–22 and 10 days, respectively); however, the median value was lower (11 days). In the first five days, 28/104 of tested *B. cereus* were detected. After 6–10 and 11–15 days, 15 and 21/104 of *B. cereus* were observed. The highest number of *B. cereus* was able to grow in the range between 16–20 days (34/104), while 6/104 were detected after 20 days of exposure at 7 °C. In the category of cereal products, including rice and seeds/pulses, *B. cereus* was detected in 58/70. Specifically, colonies from rice and pasta were detected in 7/8 and 8/10 of tested products. Further, presumptive *B. cereus* obtained from milk powder and powders containing milk or whey protein had the same ability to grow at 7 °C as at 4 °C. For colonies obtained from mashed potato flakes, instant soups, seeds, and herbs, and spices growth potential at 7 °C was slightly higher (10/10, 12/12, and 7/7, respectively). Berthold-Pluta et al. [38] tested the psychrotrophic properties of 1022 isolates obtained from different food products at 7 °C for 10 days. Results showed that 25% (256) isolates were able to grow at 7 °C, which is in line with our study where 21/104 (20.2%) grew at 7 °C in 10 days. However, the majority of tested *B. cereus* communities started to grow after 10 days. Exposure time in our study was longer; therefore we could observe more psychrotrophs. Similar results were reported for isolates obtained from refrigerated dairy products. Four out of 15 isolates (26.7%) were detected at 7 °C after 10 days of exposure. Carlin et al. [61] reported that 41/83 (49%) diarrheal and food–environment strains grew at 7 °C after 12 days of exposure. Among the 101 isolated *B. cereus* from green lettuce, only 18 were capable of growing at 7 °C [59].

Results obtained at 10 °C indicate that all presumptive *B. cereus* exhibit mesophilic character considering that 121/121 were able to grow at this temperature. On the 16th day, presumptive *B. cereus* were detected in all tested samples, with an average of seven days and a median value of four days. For the near half of the inoculated agar plates (45%), the GDT was in the first five days. After, 21 and 19/121 were detected to grow between 6–10 and 11–15 days. Other studies also reported that many *B. cereus* can grow at 10 °C. Samapundo et al. [47] detected the majority of the isolates (87.9%) during 45 days of exposure. Isolates obtained from dairy products also displayed psychrotrophic character (12/15) [68]. 

Temperature profiling of food isolates represents an important factor for food safety and quality. Determination of potential growth or toxin production at a given temperature may provide information about behavior in a specific food product that is directly related to food storage recommended temperature. Especially, spore formers are a significant risk in the case of temperature/time storage abuse. Although they grow very slowly at 4 and 7 °C; at 10 °C, psychrotrophs can grow fast and may produce toxins. 

### 2.3. Toxigenic Profiles of Psychrotrophic Presumptive B. cereus Grown at 4 °C

**Figure 1:**
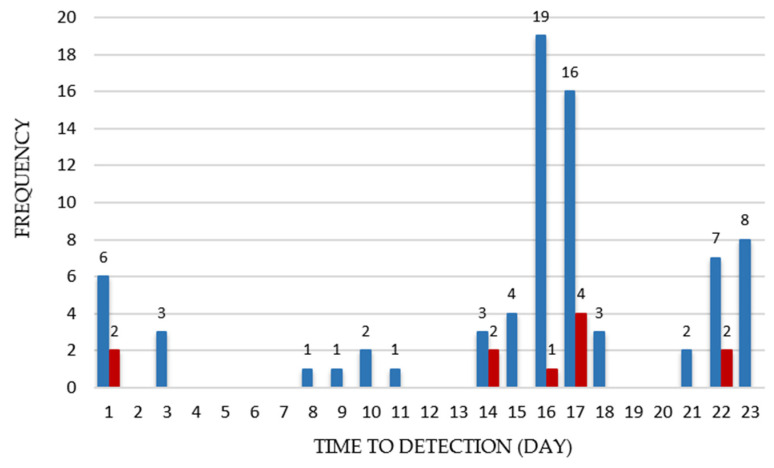
Frequency Distributions of the Time to Detection (GDT, day) of Enterotoxigenic (*n* = 76) and Emetic (*n* = 11) Psychrotrophic Presumptive *B. cereus*. Blue columns represent the number of enterotoxigenic *B. cereus*, and the orange columns represent the number of emetic *B. cereus*.

Several studies have shown that psychrotrophic *B. cereus* carry toxin encoding genes associated with both diarrheal and emetic disease. To assess the potential hazard of psychrotrophic presumptive *B. cereus* that are able to grow at refrigerated temperatures (*n* = 94), their toxigenic profiles were determined. Enterotoxin and cereulide encoding genes were detected in 81% (76/94) of psychrotrophic presumptive *B. cereus* biomasses isolated from food. The *nheA* gene was the most prevalent at 70.2% (66/94), followed the by *hblA* gene at 55% (52/94), and the *ces* encoding gene at 11.7% (11/94). None of the psychrotrophic presumptive *B. cereus* were cytotoxin K-1 encoding gene (*cytK-1*) positive. The main carrier of this gene is *B. cytotoxicus* species, which has been characterized by thermotolerant growth affinities. Presumably, ribosomes in thermophilic bacteria are non-functional at lower temperatures [69]; therefore, the absence of this microorganism among psychrotrophs was expected. Park et al. [59] reported that 94% of the psychrotolerant *B. cereus* isolates obtained from green lettuce harbored the *nheABC* genes, 44% *hbl*, while *ces* was not detected. Similarly, Bartoszewicz et al. [70] reported that 85 and 30% of psychrotrophic isolates were *nheA*, *hblA* positive, respectively. Together, *hblA* and *nheA* were present in 40% (38/94) of *B. cereus* communities that were able to grow at 4 °C, while the combination of *hblA* and *ces* was not noted. *hblA*, *nheA*, and *ces* psychrotrophic presumptive *B. cereus* were found together in 5.3% (5/94) of tested food products. Our data are consistent with previous reports demonstrating that enterotoxin encoding genes are prevalent among psychrotrophic *B. cereus*. Simultaneously, *ces* and *nheA* were detected in 7.44% (7/94), while *ces* was found alone only in one sample. Frequency distributions of the growth detection time (GDT, day) of enterotoxigenic and emetic psychrotrophic presumptive *B. cereus* are shown in Figure 1.

Among 52 presumptive *B. cereus* isolated from the category cereal products, including rice and seeds/pulses, *hblA, nheA*, and *ces* genes were detected in 34.5% (20/58), 37.9% (22/58), and 8.6% (5/58). In 27.6.% (16/58) of tested psychrotrophs, *hblA* and *nheA* were detected together, and *nheA* and *ces* in 8.6% (5/58). A combination of *hblA*, *nheA*, and *ces* was found in 7.7% (4/52). Specifically, bulgur (2/3) was contaminated with all potential toxin-producers that were able to grow at 4 °C, as well as couscous (1/5) and muesli (1/5), indicating that this type of food can be a significant source of both enterotoxigenic and emetic *B. cereus* at refrigerator temperatures. *ces* encoding gene in combination with *nheA* was found in communities obtained from buckwheat (1/2), muesli (1/5), and pasta (1/11). Further, a combination of *hblA* and *nheA* was found in millet (1/2), polenta (3/3), semolina (2/4), muesli (1/5), flour (3/12), and rice (4/8). *hblA* gene was rarely found alone-only in millet (1/2), flour (1/12), and semolina (1/4). *nheA* alone was found in oat flakes (1/5), rice waffles (1/3), flour (1/12), pasta (1/10), and rice (1/8). Seeds were contaminated with *nheA*, and *hblA*, positive *B. cereus* (3 and 2/6 samples, respectively). Dairy-based powdered products such as milk powder and derivatives of milk powder were also contaminated with psychrotrophic toxigenic *B. cereus* (9/16). From 2/16 tested products, a combination of *hblA, nheA,* and *ces* was found in milk powder (1/7) and coffee creamer (1/6). *hblA* and *nheA* were found in communities obtained from milk powder (1/6) and coffee creamer (2/6). *nheA* alone was detected in the sample obtained from chantilly cream (1/3), as well as the *ces* gene (1/3).

According to results obtained in this study, mashed potato flakes seem like the significant carriers of psychrotrophic enterotoxigenic *B. cereus*. Five out of nine psychrotrophic presumptive *B. cereus* were both *hblA* and *nheA* positive, while one out of nine was only *nheA* positive. Similarly, instant soups showed as a great source of toxigenic psychrotrophic *B. cereus* considering that 10/12 psychrotrophic communities were positive on some of the tested genes. Eight out of twelve were *hblA* and *nheA* positive, one out of twelve was *ces* and *nheA* positive. In one sample *nheA* positive psychrotrophic *B. cereus* were detected. Further, herbs and spices were contaminated with enterotoxigenic *B. cereus* (five out of six). While not many studies were conducted to examine toxigenic properties of psychrotrophic *B. cereus* isolated from specific food groups, concerns have been raised for many years due to discrepancies among data. Investigations related to foodborne outbreaks did not report properly chilled food as the main cause of *B. cereus* poisoning [29]. At low temperatures, *B. cereus* proliferates slowly; however, the effects of the food matrix seem to play an important role in toxin production. 

### 2.4. Cereulide Production by Psychrotropic Presumptive B. cereus at 37 °C and 4 °C

Cereulide producers are characterized by mesophilic and psychrotrophic growth capacities [68]. Psychrotrophs mainly belong to the *B. weihenstephanensis* species. However, the potential for psychrotrophic *B. cereus* group strains to acquire the *ces* genes by horizontal gene transfer exists. Therefore it has been assumed that potential cereulide psychrotrophic might not all belong strictly to this species [29]. In our study, we identified potential cereulide producers that can grow at refrigerator temperatures with the aim to examine the ability to produce cereulide. Presumptive psychrotrophic *B. cereus* identified as *ces* gene positive were tested by the computer-aided semen analysis study of the boar semen motility described by Rajkovic et al. [69]. After exposure to 4 °C, 11 out of 38 examined *ces* positive *B. cereus* were detected to grow. These *B. cereus* were isolated from the following food products: buckwheat, chantilly cream, coffee creamer, couscous, milk powder, muesli, polenta, instant vermicelli soup, and instant meat soup (Table 3). 

Based on the motility assay, it was seen that 5/11 of ces-positive presumptive *B. cereus* were able to produce cereulide at 37 °C. Our results are consistent with the study of Biest-Pieters et al. [39], who reported that emetic strains do not always display their pathogenic potential. Food products from which psychrotophic cereulide producers were obtained are buckwheat, chantilly cream, coffee creamer, muesli, and instant vermicelli soup. Further, to estimate the ability to produce cereulide in small quantities at 4 °C, we used LC-MS^2^. None of the *ces*-positive samples were able to produce any significant amounts of cereulide at 4 °C after one month of exposure.

Only in one sample 0.79 ng/mL of cereulide was quantified. Limited data are available about cereulide production at refrigerator temperatures. It has been noted that at 8 °C emetic strains produce minimum amounts of toxin [29,71]. This can explain why correctly stored products were never reported as a cause of intoxication with cereulide. However, it should not be ignored that, in many cases, food products are subjected to a temperature that often surpasses recommended refrigerator temperatures and, in numerous cases, can be higher than 10 °C. Cereulide synthesis is a multifactorial process that is not strictly dependent on the cell number. Present nutrients can trigger production even before the exponential growth phase [72]. Moreover, cereulide at low temperatures can be produced in high amounts [73] or in more potent forms (isocereulide A) [74,75].

## 3. Conclusions

This study demonstrated that selected food ingredients and products were significantly contaminated with toxigenic *B. cereus*. Many of them were concomitantly contaminated with different potentially pathogenic *B. cereus* gene carriers, indicating that one product may pose a risk for both diarrheal and emetic disease. The relatively high number of isolated psychrotrophic presumptive *B. cereus* were enterotoxin and cereulide gene carriers, indicating that refrigeration cannot ensure complete growth prevention. Due to the increasing number of foodborne outbreaks caused by *B. cereus* toxins, it is necessary to monitor the presence of this pathogen among food products more regularly. Moreover, in order to comply with an appropriate level of consumers protection, relevant parties such as food business operators and health authorities must take into account risks present during food handling. Since toxigenic psychrotrophic *B. cereus* is abundant in food products and ingredients, it is important to estimate possible risks related to the refrigerator and temperature abuse conditions in regards to both enterotoxin and cereulide producing *B. cereus*. Therefore, additional studies on factors that affect *B. cereus* growth and toxin production are needed.

## 4. Materials and Methods

### 4.1. Collection of Food Products

A total of 250 food products were collected from Belgian, Dutch, and Serbian retail markets. Selected products are mainly used as ingredients in mixed food products (*n* = 250). Products were classified into five categories: ‘cereal products, including rice and seeds/pulses’, ‘dairy-based products’, ‘dehydrated vegetables, ‘mixed food’, ‘herbs and spices’ according to the Manual for reporting on food-borne outbreaks in accordance with Directive 2003/99/EC published by EFSA (Table 4) [76]. Individual products within group were selected from different brands available in retail shops during 2019 and stored at recommended conditions prior to analysis. Analysis was performed a maximum of one day after purchase of ready-to-eat products and sprouted seeds or five days upon arrival of dry food products in the Laboratory of Food Microbiology and Food Preservation (Ghent University). 

### 4.2. Detection of Presumptive B. cereus

Detection of presumptive *B. cereus* was performed as previously described [47]. In brief, 25 g of selected food product was placed in a sterile filter stomacher and enrichment broth composed of Tryptone Soy Broth (TSB) (Oxoid, UK) and 50,000 IU of polymyxin B supplement (Oxoid, Hampshire, UK) was added to the total of 250 g. Enrichment of homogenized samples was performed at 30 °C for 24 h, after which 0.1 mL was inoculated onto a mannitol-egg yolk-phenol-red polymyxin-agar medium (Oxoid, Hampshire, UK), supplemented with polymyxin B according to manufacturer instructions and incubated at 30 °C for 48 h. 

### 4.3. Toxin Gene Profiling

DNA isolation was performed using Prepman lysis solution (Applied Biosystems, Waltham, MA, USA). Fresh culture of presumptive *B. cereus* was collected and placed in a lysis buffer in 2 mL tube, mixed thoroughly, and incubated at 95 °C for 15 min. Afterwards, supernatants with isolated DNA were stored at −20 °C and screened for the presence of enterotoxin and cereulide encoding genes using primers as previously described (Table 5), namely the *nheA*, *hblA*, *cytK-1*, and cereulide toxin-related genes *ces*. The specificity of used primers was primarily tested in Basic Local Alignment Search Tool Genomic (https://blast.ncbi.nlm.nih.gov/Blast.cgi (5 April 2019). Further, they were checked by testing 30 reference strains with known toxigenic properties obtained from the culture collection of Laboratory of Food Microbiology and Food Preservation (Ghent University). Genomic DNA extracted from strains ATCC 14579, DSM 4384, NVH 1230-88, NCTC 11145, NS115, NS117, LMG 12334, F4346/75, NVH0075/95, NVH0500/00, F0285/78 and NVH0391-98 were used as positive or negative controls in this study (also for cereulide production in Section 4.5), depending on their toxigenic properties, while the blank control was done using nuclease-free water.

A typical 50 μL PCR mixture for the SYBR green I real-time PCR assay consisted of 2 μL of template DNA, 25 μL of 2x concentrated PowerUp SYBR Green Master Mix (Thermo Fisher Scientific, Vilnius, Lithuania), 500 nM of reverse and forward primers (IDT Technologies, Leuven, Belgium) and 18 μL of nuclease-free water (Ambion, Austin, TX, USA). Primarily, isolated DNA was exposed to 95 °C for 10 min, followed by 40 cycles of 95 °C/15 s for denaturation, and annealing and extension at temperatures shown in Table 5. In the subsequent dissociation stage for the analysis of melting curve, the temperature was raised from 60 °C to 95 °C. Further, 20 µL PCR reaction mixture for the probe-based assay contained 10 µL of GoTaq Probe qPCR (Promega, Madison, WI, USA), 500 nM of each primer, 200 nM probe, and 2 µL of the template DNA. All PCR reactions were performed and analyzed in ABI 7300 Real Time PCR system (Applied Biosystems, Waltham, MA, USA) using MicroAmp 96-well plates (Applied Biosystems, Waltham, MA, USA). For the *ces* gene detection, two pair of primers were used (Table 5). 

### 4.4. Refrigeration and Mild Abuse Temperature Exposure

Collected presumptive *B. cereus* communities, which possessed at least one virulence gene, were tested for the capability to grow at psychotropic conditions at 4, 7, and mild abuse temperature (10 °C). Prior to exposure to respective temperatures, cultures were reactivated in TSB. After, culture was transferred onto double Tryptone Soya Agar (TSA) and incubated for 30 days at 4, 7, and 10 °C. 

### 4.5. Emetic Toxin Detection via Boar Semen Motility Assay 

The procedure was performed according to Rajkovic, Uyttendaele, and Debevere [80]. Primarily, *ces*-positive *B. cereus* samples were incubated for 24 h at 37 °C, in TSB after which 1 mL of each sample was inoculated onto TSA (in triplicates), air-dried and incubated again at 30 °C for 24 h. Grown colonies were flooded with 3 mL of methanol (≥99.9%, Sigma-Aldrich, St. Louis, MI, USA) and transferred to the 500 mL Erlenmeyer flask. Methanol was added to the final dilution of 1:3 of biomass and placed in a water bath at 100 °C for 1 h. Extracted cereulide was stored in glass vials at −20 °C until analysis. Further, five microliters of cereulide extract were mixed with 195 µL of the fresh boar semen (ca. 30 million cells per mL) in a microtiter well, and then 5 µL of suspension was immediately transferred into Leja Slides (2-chambered 20 mm slide, Leja, Nieuw-Vennep, The Netherlands). The sample was considered cereulide-positive when interruption of semen motility occurred within less than 10 min. Motility was microscopically analyzed (Zeiss, AxioCam Mrm Imager A.1, Jena, Germany) with a MiniTherm stage warmer providing an optimal temperature of 37 °C. The images were examined via the AxioVision program. Experiments were performed in three repetitions.

### 4.6. Quantification of Cereulide Production at 4 °C by LC–MS² Analysis

*ces* positive samples that were able to grow at 4 °C were tested for the cereulide production at 4 °C by LC-MS^2^. After one month of exposure, we extracted cereulide as previously described (Section 4.5.) and subjected to further analysis according to Delbrassinne et al. [81]. Briefly, the LC-MS LCQ Deca-XP Plus ion trap mass analyzer (ThermoFinnigan, San Jose, CA, USA) was used for analysis. A full mass spectrum from 500 to 1300 *m*/*z* values and an MS² fragmentation mass spectrum were recorded in positive electrospray mode (ESI+). The peaks corresponding to the loss of a CO (*m*/*z* values 1125.3 and 1083.3, respectively) were always the highest peaks generated through the fragmentation. The chromatograms were smoothed thanks to a Gaussian function. 

## Figures and Tables

**Table 1:** Prevalence of Toxigenic *B. cereus* in Selected Food Products.

Food Category	Food Subcategory	Total Number	Number of Positive Samples			
			*nheA*	*hblA*	*cytK-1*	*ces*
Cereal products, including rice and seeds/pulses		162	129	108	1	33
	Cereal grains	49	39	35	-	11
	Cereal-based products and derivatives	94	66	63	-	20
	Seeds	7	6	6		
	Sprouts	12	6	4		2
Dairy-based products		26	22	20	3	10
	Milk powder and derivative products	16	13	13	3	7
	Coffee creamer	7	7	5		1
	Chantilly cream	3	2	2		2
Dehydrated vegetables	Mashed potato flakes	17	14	9	6	7
Herbs and spices	Spices	12	2	6		
	Herbal teas	8	6	4		1
Mixed food	Ready-to-eat	12	4	2		
	Instant soups	13	11	11		7
Total		226	176	162	10	58

**Table 2:** Growth Detection Time for Presumptive *B. cereus*.

Temperature (°C)	GDT (Day)									Total Number
	1–5	6–10	11–15	16–20	20–25	Average	Standard Deviation	Median	Interquartile Range	
4	16	7	11	42	18	11	8.3	15	16	94
7	28	15	21	34	6	10	6.3	11	12	104
10	54	21	19	27	-	7	3.2	4	4	121

**Table 3:** GDT and cereulide production at 37 and 4 °C of psychrotrophic *ces*-positive *B. cereus*.

Food Product	GDT at 4 °C	Cereulide Production at 37 °C (+/−)	Cereulide Production at 4 °C (ng/mL)
Buckwheat	17	+	−
Chantilly cream	1	+	−
Coffee creamer	16	+	−
Couscous	14	−	−
Couscous	14	−	
Milk powder	1	−	−
Muesli	17	−	−
Muesli	17	+	−
Polenta	17	−	−
Instant vermicelli soup	22	+	0.79
Instant soup	22	−	-

**Table 4:** Categories of Samples Analyzed in This Study.

Food Category	Subcategory	Food Product	Number of Samples
Cereal products, including rice and seeds/pulses	Cereal grains	Buckwheat	3
		Quinoa	5
		Millet	3
		Bulgur	3
		Rice	29
		Kamut	1
	Cereal-based products and derivatives	Semolina	4
		Rice waffles	7
		Breakfast cereals	10
		Couscous	5
		Polenta	6
		Oat flakes	5
		Flour	13
		Pasta and pasta-like products	50
	Seeds		7
	Sprouted seeds		12
Dairy-based products		Milk powder	8
		Powder containing milk or whey protein (derivative products)	8
		Coffee creamer	7
		Chantilly cream	3
Dehydrated vegetables		Processed potato flakes	17
Herbs and spices		Spices	12
		Herbal teas	8
Mixed food	Ready-to-eat	Pasta salads	8
		Rice salads	4
	Soups	Instant soups	13
Total			250

**Table 5:** Primer Pairs and Annealing Temperature Conditions.

**Genes**	Primers	Annealing Temperature (°C)	Reference
*hblA*_Taq	F: ATT AAT ACA GGG GAT GGA GAA ACT T R: TGA TCC TAA TAC TTC TTC TAG ACG CTT P: FAM/TGACTGCAA/ZEN/GAG CTCTTTATT	52	[77]
*nheA*_SYBR_G	F: TTC AAA TTC AAA AGA ATG TTG AAG AAG G R: GAT TTG TTT GCT TAT TCA TTT CAT CAC	60	[78]
*cytK1*_SYBR_G	F: GCT TTG TAT AAG CAA CTT GGA TAG R: AGC CTC TGT AAC ACC AAG C	60	
*ces*_SYBR_G	F: CAC GCC GAA AGT GAT TAT ACC AA R: CAC GAT AAA ACC ACT GAG ATA GTG	60	[79]
*ces*_Taq	F: CGC CGA AAG TGA TTA TAC CAA R: TAT GCC CCG TTC TCA AAC TG P: FAM/GGG AAA ATA ACG AGA AAT GCA/TAMRA	60	

## Data Availability

Not applicable.

## References

[1] European Food Safety Authority (EFSA), European Centre for Disease Prevention and Control (ECDC) (2016). Risks for Public Health Related to the Presence of *Bacillus cereus* and other *Bacillus* spp. including *Bacillus thuringiensis* in Foodstuffs. EFSA J..

[2] Jung M.Y., Paek W.K., Park I.S., Han J.R., Sin Y., Paek J., Rhee M.S., Kim H., Song H.S., Chang Y.H. (2010). *Bacillus gaemokensis* sp. nov., Isolated from Foreshore Tidal Flat Sediment from the Yellow Sea. J. Microbiol..

[3] Jung M.Y., Kim J.S., Paek W.K., Lim J., Lee H., Kim P., Ma J.Y., Kim W., Chang Y.H. (2011). *Bacillus manliponensis* sp. nov., a New Member of the *Bacillus cereus* Group Isolated from Foreshore Tidal Flat Sediment. J. Microbiol..

[4] Liu B., Liu G.H., Hu G.P., Cetin S., Lin N.Q., Tang J.Y., Tang W.Q., Lin Y.Z. (2014). *Bacillus bingmayongensis* sp. nov., Isolated from the Pit soil of Emperor Qin’s Terra-cotta Warriors in China. Antonie Van Leeuwenhoek Int. J. Gen. Mol. Microbiol..

[5] Miller R.A., Beno S.M., Kent D.J., Carroll L.M., Martin N.H., Boor K.J., Kovac J. (2016). *Bacillus wiedmannii* sp. nov., a Psychrotolerant and Cytotoxic *Bacillus cereus* group species Isolated from Dairy Foods and Dairy Environments. Int. J. Syst. Evol. Microbiol..

[6] Liu Y., Du J., Lai Q., Zeng R., Ye D., Xu J., Shao Z. (2017). Proposal of Nine Novel Species of the *Bacillus cereus* Group. Int. J. Syst. Evol. Microbiol..

[7] Guo J., Wang Y., Yang G., Chen Y., Zhou S., Zhao Y., Zhuang L. (2016). *Bacillus Nitroreducens* sp. nov., a Humus-Reducing Bacterium Isolated from a Compost. Arch. Microbiol..

[8] Helgason E., Økstad O.L.E.A., Caugant D.A., Mock L.E., Hegna I.D., Johansen H.A., Fouet A., Mock M., Hegna I., Kolstø A.B. (2000). One Species on the Basis of Genetic Evidence. Appl. Environ. Microbiol..

[9] Guinebretière M.H., Velge P., Couvert O., Carlin F., Debuyser M.L., Nguyen-The C. (2010). Ability of *Bacillus cereus* group strains to cause Food Poisoning Varies According to Phylogenetic Affiliation (groups I to VII) Rather Than Species Affiliation. J. Clin. Microbiol..

[10] Jovanovic J., Ornelis V.F.M., Madder A., Rajkovic A. (2021). *Bacillus cereus* Food Intoxication and Toxicoinfection. Compr. Rev. Food Sci. Food Saf..

[11] Berthold-Pluta A., Pluta A., Garbowska M. (2015). The Effect of Selected Factors on the Survival of *Bacillus cereus* in the Human Gastrointestinal Tract. Microb. Pathog..

[12] Lund T., de Buyser M., Einar P., Aliments Â.S. (2000). A New Cytotoxin from *Bacillus cereus* That May Cause Necrotic Enteritis. Mol. Microbiol..

[13] Senesi S., Ghelardi E. (2010). Production, Secretion and Biological activity of *Bacillus cereus* enterotoxins. Toxins.

[14] Ehling-Schulz M., Frenzel E., Gohar M. (2015). Food-bacteria interplay: Pathometabolism of Emetic *Bacillus cereus*. Front. Microbiol..

[15] Rajkovic A., Uyttendaele M., Dierick K., Samapundo S., Botteldoorn N., Mahillon J., Heyndrickx M. (2008). Risk Profile of the *Bacillus cereus* Group Implicated in Food Poisoning. Rep. Super. Health Counc. Belg..

[16] Mahler H., Pasi A., Kramer J.M., Schulte P., Scoging A.C., Bär W., Krähenbühl S. (1997). Fulminant Liver Failure in Association with the Emetic Toxin of *Bacillus cereus*. N. Engl. J. Med..

[17] Vangoitsenhoven R., Rondas D., Crèvecoeur I., D’Hertog W., Baatsen P., Masini M., Van der Schueren B. (2014). Foodborne Cereulide Causes beta-cell Dysfunction and Apoptosis. PLoS ONE.

[18] Dierick K., van Coillie E., Swiecicka I., Meyfroidt G., Devlieger H., Meulemans A., Hoedemaekers G., Fourie L., Heyndrickx M., Mahillon J. (2005). Fatal Family Outbreak of *Bacillus cereus*. Assoc. Food Poisoning.

[19] Bauer T., Sipos W., Stark T.D., Käser T., Knecht C., Brunthaler R., Saalmüller A., Hofmann T., Ehling-Schulz M. (2018). First Insights Into within Host Translocation of the *Bacillus cereus* Toxin Cereulide Using a Porcine Model. Front. Microbiol..

[20] Naranjo M., Denayer S., Botteldoorn N., Delbrassinne L., Veys J., Waegenaere J., Sirtaine N., Driesen R.B., Sipido K.R., Mahillon J. (2011). Sudden Death of a Young Adult Associated with *Bacillus cereus* Food Poisoning. J. Clin. Microbiol..

[21] Shiota M., Saitou K., Mizumoto H., Matsusaka M., Agata N., Nakayama M., Kage M., Tatsumi S., Okamoto A., Yamaguchi S. (2010). Rapid Detoxification of Cereulide in *Bacillus cereus* Food Poisoning. Pediatrics.

[22] European Food Safety Agency (EFSA) (2005). Opinion of the Scientific Panel on Biological Hazards on *Bacillus cereus* and Other *Bacillus* spp. in Foodstuffs. EFSA J..

[23] Schreiber N., Hackl G., Reisinger A.C., Zollner-schwetz I., Eller K., Schlagenhaufen C., Pietzka A., Czerwenka C., Stark T.D., Kranzler M. (2022). Acute Liver Failure after Ingestion of Fried Rice Balls: A Case Series of *Bacillus cereus* Food Poisonings. Toxins.

[24] European Food Safety Authority (EFSA), European Centre for Disease Prevention and Control (ECDC) (2016). The European Union Summary Report on Trends and Sources of Zoonoses, Zoonotic Agents and Food-Borne outbreaks in 2015. EFSA J..

[25] European Food Safety Authority (EFSA), European Centre for Disease Prevention and Control (ECDC) (2015). The European Union Summary Report on Trends and Sources of Zoonoses, Zoonotic Agents and Food-Borne Outbreaks in 2014. EFSA J..

[26] European Food Safety Authority (EFSA), European Centre for Disease Prevention and Control (ECDC) (2017). The European Union Summary Report on Trends and Sources of Zoonoses, Zoonotic Agents and Food-Borne Outbreaks in in 2016. EFSA J..

[27] European Food Safety Authority (EFSA), European Centre for Disease Prevention and Control (ECDC) (2019). The European Union One Health 2018 Zoonoses report. EFSA J..

[28] European Food Safety Authority (EFSA), European Centre for Disease Prevention and Control (ECDC) (2021). The European Union One Health 2019 Zoonoses Report 2020. EFSA J..

[29] Webb M.D., Barker G.C., Goodburn K.E., Peck M.W. (2019). Risk Presented to Minimally Processed Chilled Foods by Psychrotrophic *Bacillus cereus*. Trends Food Sci. Technol..

[30] European Food Safety Authority (EFSA), European Centre for Disease Prevention and Control (ECDC) (2012). The European Union Summary Report on Trends and Sources of Zoonoses, Zoonotic Agents and Food-Borne Outbreaks in 2010. EFSA J..

[31] European Food Safety Authority (EFSA), European Centre for Disease Prevention and Control (ECDC) (2018). The European Union Summary Report on Trends and Sources of Zoonoses, Zoonotic Agents and Food-Borne Outbreaks in 2017. EFSA J..

[32] James C., Onarinde B.A., James S.J. (2017). The Use and Performance of Household Refrigerators: A Review. Compr. Rev. Food Sci. Food Saf..

[33] Jovanovic J., Djekic I., Smigic N., Tomic N., Rajkovic A. (2022). Temperature Profile and Hygiene in Household Refrigerators in Belgrade, Serbia and Their Relation to Consumers Food Safety Knowledge and Characteristics of the Refrigerators. Food Control.

[34] European Food Safety Authority (EFSA), European Centre for Disease Prevention and Control (ECDC) (2014). The European Union Summary Report on Trends and Sources of Zoonoses, Zoonotic Agents and Food-Borne Outbreaks in 2012. EFSA J..

[35] European Food Safety Authority (EFSA), European Centre for Disease Prevention and Control (ECDC) (2013). Analysis of the Baseline Survey on the Prevalence of *Listeria monocytogenes* in Certain Ready-to-Eat Foods in the EU, 2010–2011 Part A: *Listeria Monocytogenes* Prevalence Estimates. EFSA J..

[36] Forghani F., Kim J.B., Oh D.H. (2014). Enterotoxigenic Profiling of Emetic Toxin- and Enterotoxin-Producing *Bacillus cereus*, isolated from Food, Environmental, and Clinical Samples by Multiplex PCR. J. Food Sci..

[37] Wijnands L.M., Dufrenne J.B., Rombouts F.M., in’t Veld P.H., van Leusden F.M. (2006). Prevalence of Potentially Pathogenic *Bacillus cereus* in Food Commodities in The Netherlands. J. Food Prot..

[38] Berthold-pluta A., Pluta A., Garbowska M. (2019). Prevalence and Toxicity Characterization of *Bacillus cereus* in Food Products from Poland. Foods.

[39] Biesta-Pieters E.G., Dissel S., Reij M.W., Zwietering M.H., In’t Veld P.H. (2016). Characterization and Exposure Assessment of Emetic *Bacillus cereus* and Cereulide Production in Food Products on the Dutch Market. J. Food Prot..

[40] Böhm M., Huptas C., Krey V.M., Scherer S. (2015). Massive Horizontal Gene Transfer, Strictly Vertical Inheritance and Ancient Duplications Differentially Shape the Evolution of *Bacillus cereus* Enterotoxin Operons *hbl*, *cytK* and *nhe*. BMC Evol. Biol..

[41] Fiedler G., Schneider C., Igbinosa E.O., Kabisch J., Brinks E., Becker B., Stoll D.A., Cho G.S., Huch M., Franz C.M.A.P. (2019). Antibiotics Resistance and Toxin Profiles of *Bacillus cereus*-Group Isolates from Fresh Vegetables from German Retail Markets. BMC Microbiol..

[42] Kim B., Bang J., Kim H., Kim Y., Kim B., Beuchat L.R., Ryu J. (2014). *Bacillus cereus* and *Bacillus thuringiensis* Spores in Korean Rice: Prevalence and Toxin Production as Affected by Production Area and Degree of Milling. Food Microbiol..

[43] Glasset B., Herbin S., Guillier L., Cadel-Six S., Vignaud M., Grout J., Pairaud S., Michel V., Hennekinne J., Ramarao N. (2016). *Bacillus cereus*-Induced Food-Borne Outbreaks in France, 2007 to 2014: Epidemiology and Genetic Characterisation. Eurosurveillance.

[44] Burtscher J., Etter D., Biggel M., Schlaepfer J., Johler S. (2021). Further Insights Into the Toxicity of *Bacillus cytotoxicus* Based on Toxin Gene Profiling and Vero Cell Cytotoxicity Assays. Toxins.

[45] Cairo J., Gherman I., Day A., Cook P.E. (2022). *Bacillus cytotoxicus*—A Potentially Virulent Food-Associated Microbe. J. Appl. Microbiol..

[46] Gamage N.W., Bamforth J., Ashfaq T., Bernard K., Gräfenhan T., Walkowiak S. (2021). Profiling of *Bacillus cereus* on Canadian Grain. PLoS ONE.

[47] Samapundo S., Heyndrickx M., Xhaferi R., Devlieghere F. (2011). Incidence, Diversity and Toxin Gene Characteristics of *Bacillus cereus* Group Strains Isolated from Food Products Marketed in Belgium. Int. J. Food Microbiol..

[48] Ankolekar C., Rahmati T., Labbé R.G. (2009). Detection of Toxigenic *Bacillus cereus* and *Bacillus thuringiensis* Spores in U.S. Rice. Int. J. Food Microbiol..

[49] Koné K.M., Douamba Z., De Halleux M., Bougoudogo F., Mahillon J. (2019). Prevalence and Diversity of the Thermotolerant Bacterium *Bacillus cytotoxicus* Among Dried Food Products. J. Food Prot..

[50] Yu S., Yu P., Wang J., Li C., Guo H., Liu C., Kong L., Yu L., Wu S., Lei T. (2020). A Study on Prevalence and Characterization of *Bacillus cereus* in Ready-to-Eat Foods in China. Front. Microbiol..

[51] Rana N., Panda A.K., Pathak N., Gupta T., Thakur S.D. (2020). *Bacillus cereus*: Public Health Burden Associated with Ready-to-Eat Foods in Himachal Pradesh, India. J. Food Sci. Technol..

[52] Dufrenne J., Tatini S., Notermans S. (1994). Stability of Spores of *Bacillus cereus* Stored on Silicagel. Int. J. Food Microbiol..

[53] Messelhäusser U., Frenzel E., Blöchinger C., Zucker R., Kämpf P., Ehling-Schulz M. (2014). Emetic *Bacillus cereus* are More Volatile than Thought: Recent Foodborne Outbreaks and Prevalence Studies in Bavaria (2007–2013). BioMed Res. Int..

[54] Becker H., Schaller G., von Wiese W., Terplan G. (1994). *Bacillus cereus* in Infant Foods and Dried Milk Products. Int. J. Food Microbiol..

[55] Tatsinkou Fossi B., Tatah Kihla Akoachere J.F., Nchanji G.T., Wanji S. (2017). Occurrence, Heat and Antibiotic Resistance Profile of *Bacillus cereus* Isolated from Raw Cow and Processed Milk in Mezam Division, Cameroon. Int. J. Dairy Technol..

[56] Pei X., Yang S., Zhan L., Zhu J., Song X., Hu X., Liu G., Ma G., Li N., Yang D. (2018). Prevalence of *Bacillus cereus* in Powdered Infant and Powdered Follow-Up Formula in China. Food Control.

[57] Liu X.Y., Hu Q., Xu F., Ding S.Y., Zhu K. (2020). Characterization of *Bacillus cereus* in Dairy Products in China. Toxins.

[58] Heini N., Stephan R., Ehling-Schulz M., Johler S. (2018). Characterization of *Bacillus cereus* Group Isolates from Powdered Food Products. Int. J. Food Microbiol..

[59] Park K.M., Kim H.J. (2020). Biofilm Formation of Low-Temperature-Tolerant *Bacillus cereus* Isolated from Green Leaf Lettuce in the Cold Chain. Foods.

[60] Choma C., Guinebretière M.H., Carlin F., Schmitt P., Velge P., Granum P.E., Nguyen-The C. (2000). Prevalence, Characterization and Growth of *Bacillus cereus* in Commercial Cooked Chilled Foods Containing Vegetables. J. Appl. Microbiol..

[61] Carlin F., Fricker M., Pielaat A., Heisterkamp S., Shaheen R., Salkinoja Salonen M., Svensson B., Nguyen-the C., Ehling-Schulz M. (2006). Emetic Toxin-Producing Strains of *Bacillus cereus* Show Distinct Characteristics within the *Bacillus cereus* Group. Int. J. Food Microbiol..

[62] Okanlawon B.M., Ogunbanwo S.T., Okunlola A.O. (2010). Growth of *Bacillus cereus* Isolated from Some Traditional Condiments Under Different Regimens. Afr. J. Biotechnol..

[63] Jan S., Brunet N., Techer C., Le Maréchal C., Koné A.Z., Grosset N., Cochet M.F., Gillard A., Gautier M., Puterflam J. (2011). Biodiversity of Psychrotrophic Bacteria of the *Bacillus cereus* Group Collected in Farm and Egg Product Industry. Food Microbiol..

[64] Carroll L.M., Cheng R.A., Wiedmann M., Kovac J. (2021). Keeping Up with the *Bacillus cereus* Group: Taxonomy Through the Genomics Era and Beyond. Crit. Rev. Food Sci. Nutr..

[65] Merzougui S., Cohen N., Grosset N., Gautier M., Lkhider M. (2013). Enterotoxigenic Profiles of Psychrotolerant and Mesophilic Strains of the *Bacillus cereus* Group Isolated from Food in Morocco. Int. J. Eng. Res. Appl..

[66] Stenfors L.P., Granum P.E. (2001). Psychrotolerant Species from the *Bacillus cereus* Group are not Necessarily *Bacillus weihenstephanensis*. FEMS Microbiol. Lett..

[67] Godič Torkar K., Seme K. (2009). Antimicrobial Susceptibility, β-Lactamase and Enterotoxin Production in *Bacillus cereus* Isolates from Clinical and Food Samples. Folia Microbiol..

[68] Montanhini M.T.M., dos Santos Bersot L. (2013). Avaliação do Comportamento Psicrotrófico e Atividade Lipolítica e Proteolítica de *Bacillus cereus* Isolado de Produtos Lácteos Refrigerados. Acta Sci. Technol..

[69] Fiedoruk K., Drewnowska J.M., Daniluk T., Leszczynska K., Iwaniuk P., Swiecicka I. (2017). Ribosomal Background of the *Bacillus cereus* Group Thermotypes. Sci. Rep..

[70] Bartoszewicz M., Bideshi D.K., Kraszewska A., Modzelewska E., Swiecicka I. (2009). Natural Isolates of *Bacillus thuringiensis* Display Genetic and Psychrotrophic Properties Characteristic of *Bacillus weihenstephanensis*. J. Appl. Microbiol..

[71] Thorsen L., Budde B.B., Henrichsen L., Martinussen T., Jakobsen M. (2009). Cereulide Formation by *Bacillus Weihenstephanensis* and Mesophilic Emetic *Bacillus cereus* at Temperature Abuse Depends on Pre-Incubation conditions. Int. J. Food Microbiol..

[72] Ellouze M., Buss Da Silva N., Rouzeau-Szynalski K., Coisne L., Cantergiani F., Baranyi J. (2021). Modeling *Bacillus cereus* Growth and Cereulide Formation in Cereal-, Dairy-, Meat-, Vegetable-Based Food and Culture Medium. Front. Microbiol..

[73] Finlay W.J.J., Logan N.A., Sutherland A.D. (2000). *Bacillus cereus* Produces Most Emetic toxin at Lower Temperatures. Lett. Appl. Microbiol..

[74] Kranzler M., Stollewerk K., Rouzeau-szynalski K., Blayo L., Sulyok M., Ehling-Schulz M. (2016). Temperature Exerts Control of *Bacillus cereus* Emetic Toxin Production on Post-Transcriptional Levels. Front. Microbiol..

[75] Marxen S., Stark T.D., Frenzel E., Rütschle A., Lücking G., Pürstinger G., Pohl E.E., Scherer S., Ehling-Schulz M., Hofmann T. (2015). Chemodiversity of Cereulide, the Emetic Toxin of *Bacillus cereus*. Anal. Bioanal. Chem..

[76] European Food Safety Agency (EFSA) (2017). Manual for Reporting on Food-Borne Outbreaks in Accordance with Directive 2003/99/EC for Information Deriving from the Year 2016. EFSA Support. Publ..

[77] Yang I.C., Shih D.Y.C., Huang T.P., Huang Y.P., Wang J.Y., Pan T.M. (2005). Establishment of a Novel Multiplex PCR Assay and Detection of Toxigenic Strains of the Species in the *Bacillus cereus* Group. J. Food Prot..

[78] Wehrle E., Didier A., Moravek M., Dietrich R., Märtlbauer E. (2010). Detection of *Bacillus cereus* with Enteropathogenic Potential by Multiplex Real-Time PCR-Based on SYBR Green I. Mol. Cell. Probes.

[79] Fricker M., Messelhäußer U., Busch U., Scherer S., Ehling-Schulz M. (2007). Diagnostic Real-Time PCR Assays for the Detection of Emetic *Bacillus cereus* Strains in Foods and Recent Food-Borne Outbreaks. Appl. Environ. Microbiol..

[80] Rajkovic A., Uyttendaele M., Debevere J. (2007). Computer Aided Boar Semen Motility Analysis for Cereulide Detection in Different Food Matrices. Int. J. Food. Microl..

[81] Delbrassinne L., Andjelkovic M., Rajkovic A., Dubois P., Nguessan E., Mahillon J., van Loco J. (2012). Determination of *Bacillus cereus* Emetic Toxin in Food Products by Means of LC-MS^2^. Food Anal. Methods.

